# Inhibitions and Down-Regulation of Motor Protein Eg5 Expression in Primary Sensory Neurons Reveal a Novel Therapeutic Target for Pathological Pain

**DOI:** 10.1007/s13311-022-01263-2

**Published:** 2022-06-28

**Authors:** Na Wei, Yang Yu, Yan Yang, Xiao-Liang Wang, Zhen-Juan Zhong, Xue-Feng Chen, Yao-Qing Yu

**Affiliations:** 1 Institute for Biomedical Sciences of Pain, Tangdu Hospital, The Fourth Military Medical University, 569 Xinsi Road, Baqiao, Xi’an, 710038 China; 2 Key Laboratory of Brain Stress and Behavior, People’s Liberation Army, Xi’an, 710038 China

**Keywords:** Eg5, VR1, Virus, DRG, Spinal cord, Pain

## Abstract

The motor protein Eg5, known as kif11 or kinesin-5, interacts with adjacent microtubules in the mitotic spindle and plays essential roles in cell division, yet the function of Eg5 in mature postmitotic neurons remains largely unknown. In this study, we investigated the contribution and molecular mechanism of Eg5 in pathological pain. Pharmacological inhibition of Eg5 and a specific shRNA-expressing viral vector reversed complete Freund’s adjuvant (CFA)-induced pain and abrogated vanilloid receptor subtype 1 (VR1) expression in dorsal root ganglion (DRG) neurons. In the dorsal root, Eg5 inhibition promoted VR1 axonal transport and decreased VR1 expression. In the spinal cord, Eg5 inhibition suppressed VR1 expression in axon terminals and impaired synapse formation in superficial laminae I/II. Finally, we showed that Eg5 is necessary for PI3K/Akt signalling-mediated VR1 membrane trafficking and pathological pain. The present study provides compelling evidence of a noncanonical function of Eg5 in primary sensory neurons. These results suggest that Eg5 may be a potential therapeutic target for intractable pain.

**Supplementary Information:**

The online version contains supplementary material available at 10.1007/s13311-022-01263-2.

## Introduction

Eg5, known as kif11 or kinesin-5, is an evolutionarily conserved kinesin that is essential for centrosome movement and bipolar spindle formation during cell division [1–4]. The bipolar structure of Eg5 enables the motor protein to crosslink and slide antiparallel microtubules apart, generated an outward pushing force, and promotes centrosome separation and bipolar spindle formation [5, 6]. The biological functions of Eg5 are modulated by phosphorylation [3, 7, 8] and acetylation [9] at different amino acid residues of Eg5. Eg5 inhibitors abolish spindle assembly, block mitotic progression and show anticancer effects [10, 11]. Beyond mitotic cells, Eg5 was detectable in postmitotic mature neurons, acting as a brake on microtubule movements and promoting the regeneration of injured axons [12, 13]. However, the function of Eg5 in mature neurons has not been fully explored.

The noncanonical function of Eg5 in cytokinesis and postanaphase was reported in plants [14, 15] but not in mammals. Accumulating evidence has shown the contributions of kinesin proteins (KIF17, KIF13B and KIF5B) to the membrane trafficking of pain-related molecules (NMDA receptors, VR1 and Na_V_1.8) [16–18], suggesting that kinesins might be neoteric and efficacious targets for pathological pain [19]. The molecular mechanisms of Eg5 in pain modulation remain incompletely understood.

Here, we discovered a noncanonical function of the motor protein Eg5 in pathological pain. Pharmacological and shRNA virus results show that Eg5 modulates VR1 expression in dorsal root ganglion (DRG) neuronal soma, nerve fibres and central terminals as well as synapse formation in the dorsal horn. We further found that Eg5 contributes to pathological pain and VR1 membrane trafficking through the PI3K/Akt signalling pathway. Our results lay the groundwork for targeting Eg5 in mature postmitotic neurons in pathological pain modulation.

### Materials and Methods

#### Experimental Animals

The behavioural experiments were performed on male Sprague–Dawley albino rats (purchased from Laboratory Animal Center of FMMU) weighing 150–200 g (age > 4 postnatal weeks). The animals had access to water and food ad libitum and were maintained at room temperature (22–26 °C) with a light/dark cycle of 12 h. The number of animals used and their suffering were minimized. All animal experiments were performed in accordance with the ARRIVE guidelines and approved by the Institutional Animal Care and Use Committee of FMMU.

#### Inflammatory Pain Model and Behaviour

Complete Freund’s adjuvant (CFA, 100 μl, 1:1 dissolved in 0.9% sterile saline) was administered to the plantar surface of the rat left hindpaw to induce inflammatory pain one day after CFA injection [20]. For assessment of heat hypersensitivity, rats were placed in a plastic chamber on the surface of a 2-mm-thick glass plate, and the sensitivity to heat stimuli was detected by an RTY-3 radiant heat stimulator (Xi’an Bobang Technologies of Chemical Industry Co., Ltd., China). The heat stimuli were applied to both the injection site and the corresponding area of the contralateral paw, and the latency was determined as the duration from the beginning of heat stimuli to the occurrence of a marked withdrawal reflex. Five stimuli were repeated for each site, and the latter three values were averaged as the mean paw withdrawal thermal latency (PWTL, s).

#### Drug Delivery

Intrathecal (i.t.) and intraperitoneal (i.p.) delivery methods were previously described [20]. Monastrol (100 μM, 10 μl, Santa Cruz, USA, sc-202710A) and YS-49 (10 μM, 10 μl, Sigma, USA, Y1521) dissolved in 0.5% DMSO were administered via i.t. or i.p. delivery twice daily for three consecutive days.

#### Eg5 shRNA Virus and VR1 Overexpression Virus

Adeno-associated virus serotype 9 (AAV9) encoding shRNA was designed based on rat Eg5 (kinesin family member 11, Kif11, NM_001169112.1). AAV scramble or Eg5 shRNA virus (1 × 10^13^ viral genomes (vg)/mL) was engineered as fusions with ZsGreen fluorescence protein (Biowit Technologies, China). In the AAV9-shRNA vector, the shRNA and ZsGreen sequences were driven by the human polymerase III human U6 and CMV promoters, respectively. Eg5 primers were designed as follows: forward primer: 5'-TGGACGTTCACAAAGCACTG-3', reverse primer: 5'-GCTGCTAACGACTGCTCTTC-3’' β-actin primers were designed as follows: forward primer: 5'-CACGATGGAGGGGCCGGACTCATC-3', reverse primer: 5'-TAAAGACCTCTATGCCAACACAGT-3'.

AAV9-hSyn-VR1-mCherry virus (1 × 10^13^ vg/mL) was designed based on rat VR1 (TRPV1, NM_031982) and was engineered with CV235 vector (BamHI/AgeI) (GeneChem, China). The primer sequences used for VR1 amplification were as follows: forward primer: GGAGGTAGTGGAATGGATCCCGCCACCATGGAACAACGGGCTAGCTTAGACTCAG, reverse primer: GTTGATTATCGATAACCGGTTTACTTGTACAGCTCGTCCATGCCGCC. Transformant primers were as follows: forward primer: CAAGAGAGCAAGAACATCTG, reverse primer: AGCGTAAAAGGAGCAACATAG.

#### Virus Intraganglionic Injection

AAV virus intraganglionic injection was performed as previously described [21]. The rats were anaesthetized with sodium pentobarbital (50 mg/kg, i.p.), and a laminectomy was performed from the L4 to L5 vertebrae to expose the DRGs for intraganglionic injection (2 μl, 1 × 10^13^ viral genomes (vg)/mL, Biowit Technologies, China) into the left L4-5 DRGs. The injection needle was withdrawn 10 s after the end of the injection. Rats were allowed to recover for 3–4 weeks prior to behaviour or staining experiments.

#### Cell Culture and Virus Transfection

Cultures of dissociated DRG neurons from rats were prepared as previously described [12]. Rats were euthanized under sodium pentobarbital anaesthesia, and the spinal column was opened. Up to 10 DRGs along the vertebral column were removed under aseptic conditions and placed into Petri dishes filled with cold, oxygenated DMEM (HyClone, USA, SH30022.01). The supernatant was removed, and DRGs were enzymatically digested, dissociated, washed and resuspended at 75,000 cells/ml. Cultures were maintained for 4–6 h in DMEM with 10% bovine calf serum (Gibco, USA, 10099141C), penicillin (100 U/ml) and streptomycin (0.1 mg/ml) (HyClone, USA, SH40003.01). Cells were then cultured in neurobasal-A medium (Gibco, USA, 10,888,022) with 2% B27 (Gibco, USA, 17,504,044), 0.5 mM GlutaMAX (Gibco, USA, 35,050–061), 50 ng/ml nerve growth factor (Gibco, USA, 13,257,019), penicillin (100 U/ml) and streptomycin (0.1 mg/ml) (HyClone, USA, SH40003.01) on poly-L-lysine (Sigma, USA, P8920)-coated glass coverslips. Cultured neurons were transfected with either scramble or Eg5 shRNA virus (1 μl, 1 × 10^13^ vg/mL) in medium containing neurobasal-A, 2% B27, 0.5 mM GlutaMAX and 50 ng/ml NGF for 24 h. The culture medium was replaced with medium containing 10 μM FUDR (Sigma, USA, F0503) and 10 μM uridine (Sigma, USA, U3003).

#### Vesicle Transport and Axonal Branching

Live cell imaging in AAV9 virus-transfected DRG neurons was performed as described previously [12]. For analysis of fluorescence-positive vesicle transport, 360 time-lapse images were taken at 40 ms exposure at 5 s intervals for each axon in a live cell imaging system (Cell^R MT20, Olympus, Japan). The velocity of fluorescence-positive vesicle transport was analysed using distance/time (μm/min). For the live cell imaging of stepwise axon and branch growth, image acquisition was performed at 15-s intervals for 1 h using a heated stage apparatus to maintain the temperature at 37 °C.

#### Calcium Imaging

Cultured rat DRG neurons were loaded with 2 μM Fura-2 acetoxymethyl ester (fura-2 AM) (Sigma, USA, F0888) at 37 °C for 30 min. Cells were washed three times and incubated in calcium imaging buffer (130 mM NaCl, 3 mM KCl, 2.5 mM CaCl2, 0.6 mM MgCl2, 10 mM HEPES, 10 mM glucose, 1.2 mM NaHCO3, adjusted to pH 7.4 with NaOH) at room temperature for 30 min. Capsaicin (5 μM, Sigma, USA, M2028) and KCl (50 mM, Sinopharm, China, 10,016,318) were applied to examine the calcium imaging response. Where indicated, neurons were challenged with corresponding dilutions of vehicle, monastrol (100 μM, Santa Cruz, USA, sc-202710A), and the PI3K inhibitor LY294002 (10 μM, MCE, HY-10108). Fluorescence was recorded at excitation wavelengths of 340 nm and 380 nm at 2-s intervals using a live cell imaging system (Cell^R MT20, Olympus, Japan). Neurons were considered responsive if they demonstrated a change in the fluorescence ratio (F340/F380) > 10% of the baseline.

#### Membrane Loading with FM Dye and Photobleaching

FM dye membrane loading and photobleaching methods were modified from a previous report [22]. For loading of the membrane, DRG neurons were bathed in a solution containing 5 μM SynaptoGreen C4 (FM1-43) (Biotium, USA, 70,022) for 90 s. Regions of interest (ROIs) for measuring recovery after photobleaching were 15 μm × 15 μm in size and contained approximately a quarter of the membrane region of one DRG neuron. The standard fluorescence recovery after photobleaching (FRAP) protocol involved six prebleach images at 2-s intervals (60% laser intensity, 488 nm), one bleach iteration (100% intensity, 405 nm, 20 s) and 43 postbleach images at 1.6 s intervals (60% intensity, 488 nm). A recovery curve was obtained simultaneously by measuring the average fluorescence intensity. Fluorescence during recovery was corrected for the bleaching produced by the 60% intensity acquisition illumination, which was measured in control experiments using the same imaging protocol but omitting the intense bleaching stimulus.

#### Electron Microscopy

Electron microscopy was performed as previously described [23]. Rats were anaesthetized with sodium pentobarbital (50 mg/kg, i.p.) and perfused with 2.5% glutaraldehyde and 4% paraformaldehyde in 0.1 M phosphate buffer (pH 7.4). Tissues were dissected, cut into 2 mm × 2 mm segments and postfixed in 2.5% glutaraldehyde (SPI Supplies, USA, 02607-BA). Segments were washed in 0.1 M phosphate buffer, fixed using 1% osmium tetroxide (Ted Pella, Inc., USA, 18456), followed by dehydration with an increasing concentration gradient of ethanol and propylene oxide. The osmicated tissue blocks were embedded in Epon-812 (SPI Supplies, USA, 02659-AB). Semithin sections (1 μm) were cut by an Ultratome (EM UC7, Leica, Germany) and collected into slides. To wash the epoxy resin, we prepared a stock solution with 5 g NaOH in 50 ml of alcohol. Slides were incubated in working solution with dimethylbenzene 3:1 (v/v) for 5 min at room temperature and washed in graded ethanol and distilled water. Sections were stained in 1% toluidine blue (Sinopharm Chemical Reagent Co., Ltd., China) for 5 min, dehydrated with graded ethanol, vitrified in dimethylbenzene and then mounted under coverslips with neutral resin. Images were taken under an optical microscope (Olympus, Japan). Ultrathin sections (50 nm) were cut by an Ultratome (EM UC7, Leica, Germany) and collected by copper grids (200 mesh). The ultrathin sections were stained with uranyl acetate (Electron Microscopy China, China, GZ02625-3) and lead citrate (Electron Microscopy China, China, GZ10701-2). Images were taken using an electron microscope (TH7700, Hitachi, Japan).

#### Immunohistochemistry Staining

Immunohistochemistry staining was performed as previously described [20, 24]. The rats were anaesthetized with sodium pentobarbital (50 mg/kg, i.p.). The lumbar spinal cord and L4-6 DRGs were dissected, postfixed for 8 h and cryoprotected in 20% sucrose in PBS overnight at 4 °C. Transverse frozen Sects. (20-μm thick) were cut on a CM1900 freezing microtome (Leica, Germany), incubated for 4 h in 0.05% Triton X-100 and 10% goat serum in phosphate buffered saline (PBS) at room temperature, and incubated with primary antibodies at 4 °C overnight with agitation. After three washes with PBS, the sections were incubated with secondary antibodies for 2 h at room temperature. The following primary antibodies were used: mouse anti-Eg5 (1:200, Abcam, USA, ab151186), rabbit anti-Eg5 (phospho T926) (1:200, Abcam, USA, ab61104), rabbit anti-TRPV1 (1:200, Alomone, Israel, ACC-030), rabbit anti-P2X3 receptor (1:200, Millipore, USA, AB5895), mouse anti-neurofilament 200 (1:200, Sigma, USA, N0142) and mouse anti-synapsin Ia/b (1;200, Santa Cruz, USA, sc-376623). The secondary antibodies were Cy3-conjugated sheep anti-rabbit IgG (1:400, Sigma, USA, C2306), FITC-conjugated goat anti-rabbit IgG (1:200, Chemicon, USA, Ap307F), FITC-conjugated bovine anti-mouse IgG (1:400, Santa Cruz, USA, SC-2366), Alexa Fluor 350 donkey anti-mouse IgG (1:500, Invitrogen, USA, A10035), and Alexa Fluor 594-conjugated isolestin GS-IB4 (1:200, Thermo, USA, I21413). We used 4′,6-diamidino-2-phenylindole dihydrochloride (DAPI, 1:2000, D9542, Sigma, USA) as a cell counterstain. Photomicrographic images were obtained under a laser scan confocal fluorescence microscope (Olympus FV1000, Japan). Cells were counted by Image-Pro Plus digitizing software (Olympus, Japan).

#### Western Blot

Western blotting was performed as previously reported [20]. Total proteins from rat DRGs were extracted by homogenization in ice-cold RIPA lysis buffer (Applygen Technologies, China) containing 50 mM Tris (pH 7.4), 150 mM NaCl, 1% NP-40 and 0.1% sodium dodecyl sulphate (SDS). Protein concentrations were determined by a BCA™ protein assay kit (Thermo Scientific, USA.). Samples were heated for 10 min at 95 °C with SDS-PAGE sample buffer, and the same amounts of proteins (45 μg) were separated by 10% SDS-PAGE separation gels and subsequently transblotted onto PVDF membranes (Immobilon P, Millipore, Billerica, MA). We used rabbit anti-Eg5 (phospho-T926) (1:200, Abcam, USA, ab61104) and rabbit anti-TRPV1 (1:300, Alomone, Israel, ACC-030) as primary antibodies and horseradish peroxidase (HRP)-conjugated goat anti-rabbit IgG as the secondary antibody (1:2000, ZSGB-Bio, Beijing, China). Mouse anti-β-actin antibody (1:1000, Sigma-Aldrich, USA, A1978) was used as an internal control. The membranes were developed with an ECL chemiluminescent substrate kit, and the signals were captured with FluorChem FC2 (Alpha Innotech Corp., San Leandro, CA). Scanned images were analysed by AlphaImager EP Analysis Software (Cell Biosciences, Inc.).

#### Statistical Analysis

For behavioural experiments, *n* refers to the number of animals. For immunohistochemistry imaging data, the number of animals used is indicated in the legend. For photobleaching imaging experiments, *n* refers to the number of cells responding to any stimulus. For vesicle transport experiments, *n* refers to the number of recorded fluorescence-positive vesicles. For electron microscopy experiments, *n* refers to the synapse number of regions of interest. No sample size calculation was performed; however, our samples are similar to those used in the field. Statistical analyses were performed using GraphPad Prism Software. All data are expressed as the mean ± standard error (SEM) unless otherwise stipulated. Descriptions of the tests used and *n* are located in the figure legends. Significance was defined as follows: *ns* not significant, *P < 0.05, **P < 0.01 and ***P < 0.001.

### Results

#### Eg5 Expression Profile in DRG Neurons and the Analgesic Effects of an Eg5 Inhibitor

**Fig. 1:**
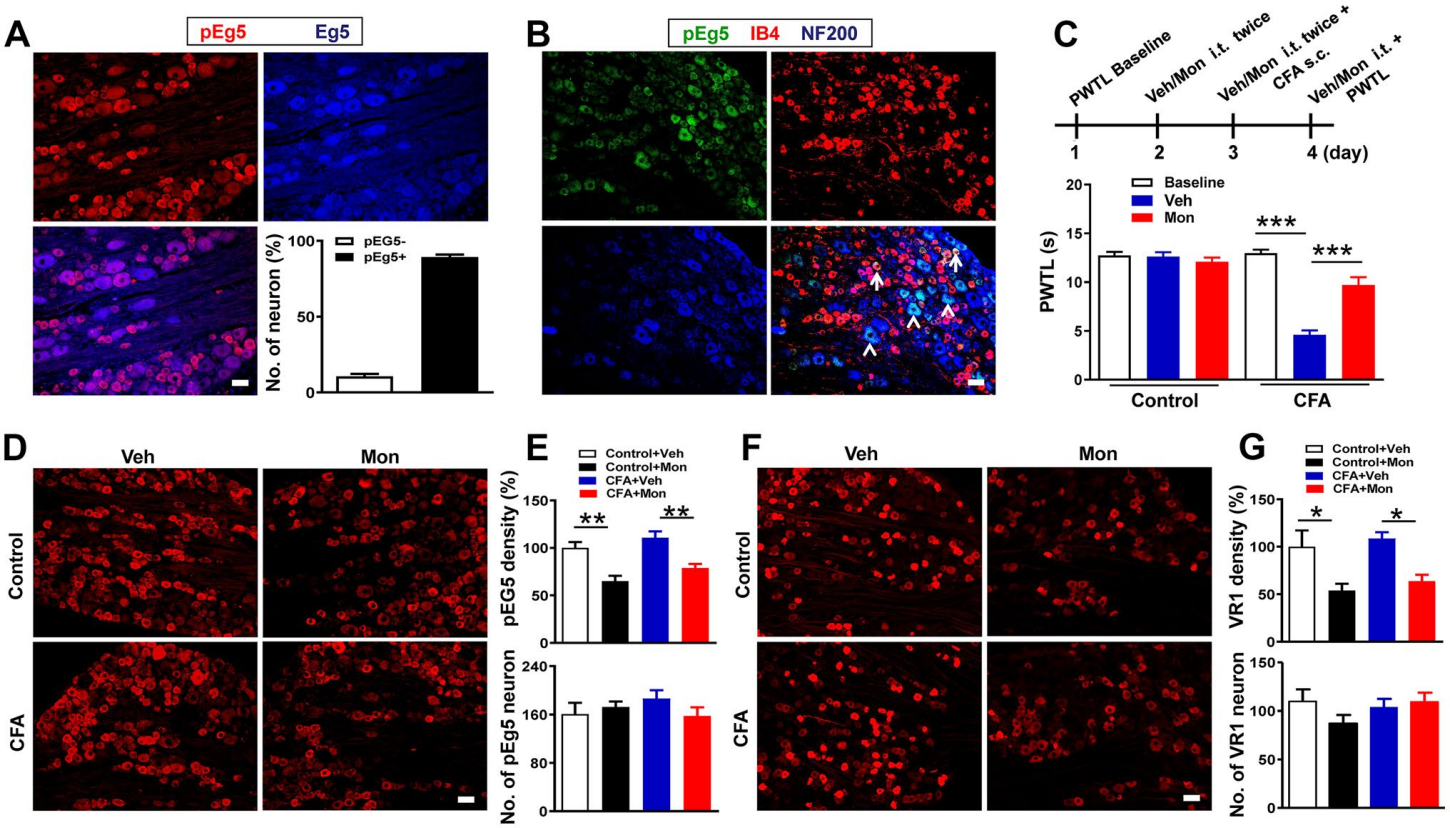
Eg5 expression in DRGs and the analgesic effect of an Eg5 inhibitor. **A** Double immunohistochemistry of Eg5 and phospho-EG5 (pEg5) in DRGs. **B** Representative immunofluorescence images of pEg5 with IB4 and NF200 in DRGs. Arrows and arrowheads indicate IB4 and NF200 neurons, respectively. **C** Schematic of the Eg5 inhibitor monastrol i.t. injection and the effects on CFA-induced inflammatory pain behaviour (*n* = 5–7 mice for each group). **D** Examples of immunofluorescence images of pEg5 in DRGs in vehicle and monastrol treatment. **E** Statistical analysis of the effects of monastrol on pEg5 density and neuron number in the control and CFA treatment groups (*n* = 3 mice and 9–11 sections for each group). **F** Examples of immunofluorescence images of VR1 in DRGs in the vehicle and monastrol treatment groups. **G** Statistical analysis of the effects of monastrol on VR1 density and neuron numbers in the control and CFA treatment groups (*n* = 3 mice and 7–10 sections for each group). Veh, vehicle control; Mon, monastrol. Scale bars, 50 μm. One-way ANOVA with Tukey’s post hoc test in (**C**), (**E**) and (**G**). *P < 0.05, **P < 0.01, ***P < 0.001. Error bars show the mean ± SEM

To reveal the expression profile of Eg5, we performed immunohistochemistry of Eg5 and phospho-EG5 (pEg5, phosphorylated at threonine 926, which is the key site for the Eg5 and microtubule interaction [3]) and found that 89% of neurons were pEg5-positive in the DRG (Fig. 1A). Triple immunofluorescence labelling showed that pEg5 colocalized with the small-sized neural marker IB4 or the large-sized neural marker NF-200 (Fig. 1B). These data prompted us to examine the roles of neuronal Eg5 in pain modulation. The Eg5-specific inhibitor monastrol (1 mM, 10 μl) was given via i.t. administration twice daily for three consecutive days. Our unpublished data showed that i.t. monastrol had no effects on motor function. The behavioural test showed that monastrol reversed CFA-induced heat hyperalgesia (9.7 ± 0.8 s versus 4.6 ± 0.5 s, *P* < 0.001) but had no effects on the control group (Fig. 1C).

We examined the effects of monastrol on pEg5 and VR1, a well-established thermal nociceptor [25, 26] (Fig. 1D). Immunohistochemistry data showed that pEg5 density, but not neuron number, was significantly reduced by monastrol (100 ± 6.2% versus 65.1 ± 5.6%, 110.9 ± 6.6% versus 78.9 ± 4.3%, *P* < 0.01, Fig. 1E). Similarly, monastrol attenuated VR1 density in the control and CFA groups (100 ± 17.1% versus 53.9 ± 7.1%, 108.6 ± 6.7% versus 63.9 ± 6.8%, *P* < 0.05, Fig. 1F, G). We observed that CFA inflammation slightly increased pEg5 and VR1 expression by 10% and 8%, respectively. However, these results did not influence our main aim of evaluating the roles of Eg5 in control and CFA-induced pain conditions.

Morphological and pharmacological results showed that Eg5 was widely expressed in primary sensory neurons and that Eg5 blockade attenuated VR1 expression and inflammatory pain behaviours. Our data indicated a previously unknown role of the motor protein Eg5 in sensory neurons for pathological pain development.

#### Eg5 shRNA Inhibits Inflammatory Pain and VR1 Expression in DRG Neurons

**Fig. 2:**
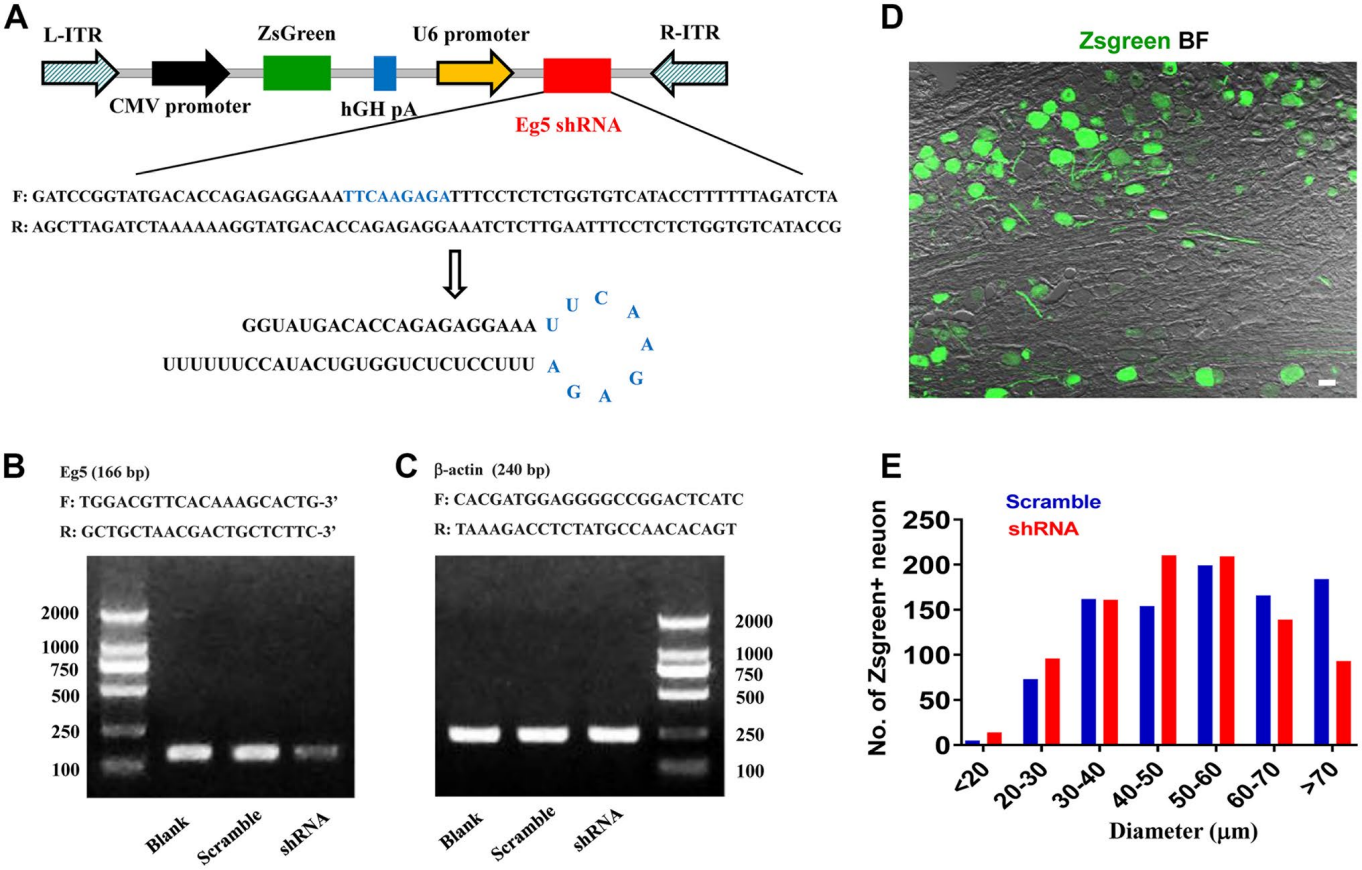
Eg5 shRNA design and identification. **A** Schematic of the targeting strategy for the Eg5 shRNA virus. **B** Gel image of the Eg5 genotype PCR results of the blank, scramble and shRNA virus groups. **C** Gel image of β-actin genotype PCR results of the blank, scramble and shRNA virus groups. **D** Representative image of ZsGreen expression in the DRG after Eg5 shRNA virus intraganglionic injection. BF, bright field. **E** ZsGreen distributions in the different sizes of DRG neurons after scramble and shRNA virus intraganglionic injections (*n* = 4 mice and 17–20 sections for each group). Scale bars, 50 μm

To fully investigate the mechanism of Eg5 in pathological pain, we generated adeno-associated virus 9 (AAV9) expressing Eg5 short hairpin-structured RNA (Eg5 shRNA) and the fluorescence protein ZsGreen (Fig. 2A). Genotype PCR results confirmed the knockdown effects of Eg5 shRNA virus (Fig. 2B, C). Immunohistochemistry results showed similar distributions of ZsGreen in DRG neurons of different sizes after scramble and shRNA virus intraganglionic injections (*n* = 4 mice and 17–20 sections for each group, Fig. 2D, E). Based on the similar transfection efficiency of scramble and shRNA, we could compare the effects of these viruses in the following studies. Notably, we found that the shRNA virus was efficiently transfected in vivo into DRG neurons (Fig. S1A) and in vitro into cultured neurons (Fig. S1B, C). These data supported the high efficiency of shRNA virus in neuronal transfection in the DRG.

**Fig. 3:**
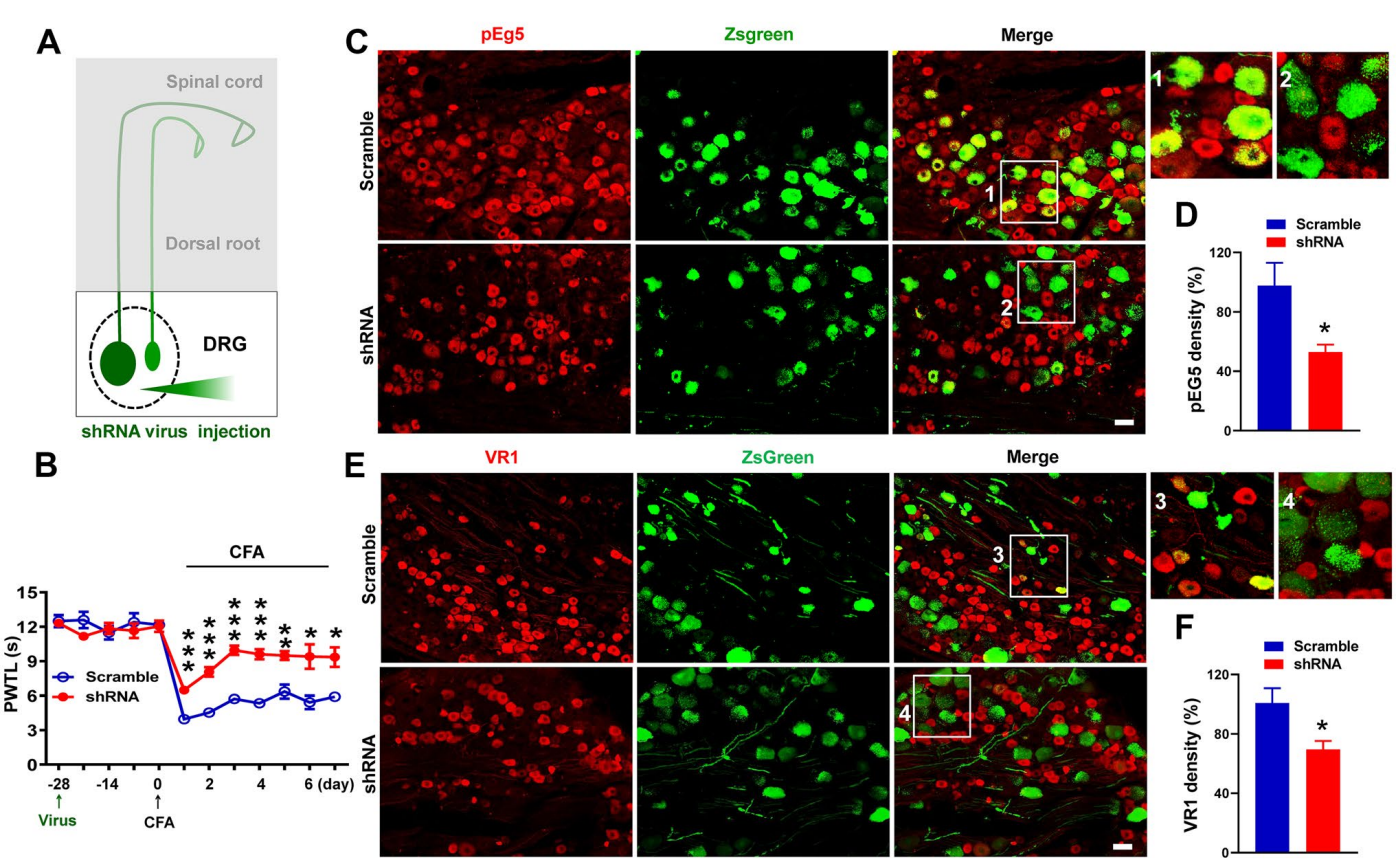
Intraganglionic injection of Eg5 shRNA virus inhibits inflammatory pain and reduces VR1 expression in the DRG. **A** Schematic of ZsGreen-labelled DRG neurons after shRNA virus intraganglionic injection. **B** Effects of Eg5 shRNA injection on CFA-induced inflammatory pain behaviour (*n* = 5–12 mice for each group). **C** Examples of immunofluorescence images of pEg5 and ZsGreen in the DRGs treated with scramble and shRNA. **D** Statistical analysis of pEg5 density in the DRGs with scramble and shRNA (*n* = 3 mice and 8–9 sections for each group). **E** Examples of immunofluorescence images of VR1 and ZsGreen in the DRGs with scramble and shRNA. **F** Statistical analysis of VR1 density in the DRGs with scramble and shRNA (*n* = 3 mice and 8–9 sections for each group). Scale bars, 50 μm. Unpaired *t* test in (**B**), (**D**) and (**F**). *P < 0.05, **P < 0.01, ***P < 0.001. Error bars show the mean ± SEM

Four weeks after intraganglionic virus injection (Fig. 3A), Eg5 shRNA suppressed the development of CFA-induced heat hyperalgesia, and the analgesic effects were maintained for at least 7 days (Fig. 3B). Immunostaining results showed that shRNA virus reduced pEg5 density in DRGs (98 ± 15.3% versus 53 ± 5.0%, *P* < 0.05) (Fig. 3C, D). Moreover, VR1 density was decreased by Eg5 shRNA (100.9 ± 9.9% versus 69.5 ± 5.8%, *P* < 0.05) (Fig. 3E, F). These results showed that intraganglionic injection of Eg5 shRNA inhibited VR1 expression in the soma of DRG neurons and reversed inflammatory pain hypersensitivity.

#### Eg5 Inhibitions Promote VR1 Axonal Transport and Decrease VR1 Expression in Dorsal Roots

**Fig. 4:**
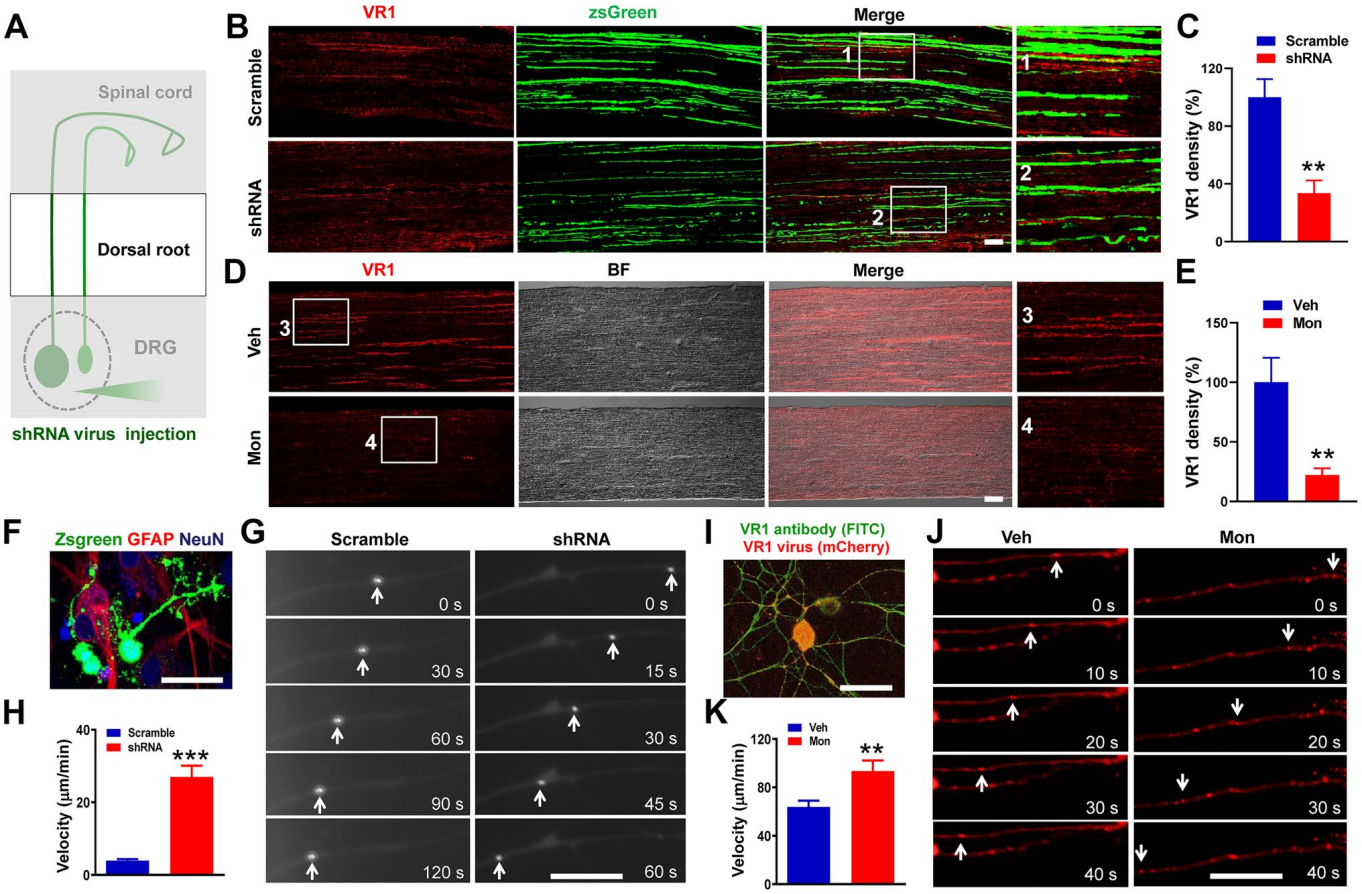
Eg5 inhibition and down-regulation reduce VR1 expression and accelerate VR1 anterograde transport in dorsal roots. **A** Schematic of ZsGreen-labelled dorsal roots after shRNA virus intraganglionic injection. **B** Examples of immunofluorescence images of VR1 staining and ZsGreen in dorsal roots with scramble and shRNA. **C** Statistical analysis of VR1 density in dorsal roots with scramble and shRNA (*n* = 3 mice and 6–7 sections for each group). **D** Examples of immunofluorescence images of VR1 in dorsal roots with vehicle and monastrol. **E** Statistical analysis of VR1 density in dorsal roots with vehicle and monastrol (*n* = 3 mice and 6 sections for each group). **F** An example of a multicoloured immunofluorescence image of neurons and glia after ZsGreen-labelled Eg5 shRNA virus transfection in DRG neuron culture. **G** Representative images of ZsGreen vesicle anterograde transport in nerve fibres with scramble and shRNA. Anterograde: towards left. **H** Statistical analysis of ZsGreen vesicle anterograde transport in nerve fibres with scramble and shRNA (*n* = 12–21 vesicles for each group). **I** Example of immunofluorescence images of VR1 antibody staining and mCherry after VR1 overexpression virus transfection in DRG neuron cultures. **J** Representative images of VR1-mCherry vesicle anterograde transport in nerve fibres with vehicle and monastrol. Anterograde: towards left. **K** Statistical analysis of VR1-mCherry vesicle anterograde transport in nerve fibres with vehicle and monastrol (*n* = 21–25 vesicles for each group). Veh, vehicle control; Mon, monastrol. Scale bars, 50 μm in (**B**), (**D**), (**F**) and (**I**) and 25 μm in (**G**) and (**J**). Unpaired *t* test in (**C**), (**E**), (**H**) and (**K**). **P < 0.05, ***P < 0.001. Error bars show the mean ± SEM

We found ZsGreen-positive fibres in DRG neurons (Figs. 2D, 3E and S1A, C), indicating the potential effects of the shRNA virus in axons. This finding prompted us to detect the effects of Eg5 shRNA in dorsal roots after intraganglionic injection (Fig. 4A). Immunohistochemistry data showed that the Eg5 shRNA virus intensively transfected dorsal roots and reduced the VR1 density (100 ± 12.5% versus 33.7 ± 8.7%, *P* < 0.01, Fig. 4B, C). The suppressive effect of Eg5 inhibition was confirmed by i.t. administration of monastrol (100 ± 20.6% versus 22.1 ± 5.5%, *P* < 0.01, Fig. 4D, E). shRNA and pharmacological results suggested that Eg5 inhibitions impaired VR1 expression in axons.

By vesicle transport assays, we found that Eg5 shRNA accelerated the average velocity of anterograde transport of the ZsGreen fluorescence protein in DRG neuronal axons (3.9 ± 0.4 versus 27 ± 3.1 μm/min, *P* < 0.001, Fig. 4F, G and H). Then, we generated an AAV9 virus containing the VR1-mCherry fusion protein (Fig. S2) and confirmed the colocalization of VR1 and the reporter protein mCherry in the soma and axon of DRG neurons (Fig. 4I and S2D). Using the VR1-mCherry virus, we directly observed VR1 transport in axons (Fig. 4J). Monastrol application increased the average velocity of VR1 anterograde transport in axons of cultured DRG neurons (63.8 ± 5.2 versus 93.5 ± 8.6 μm/min, *P* < 0.01, Fig. 4K, Movies S1–S2). Therefore, Eg5 inhibition accelerated VR1 anterograde transport in the axons of DRG neurons and impaired VR1 expression in the dorsal root of DRG neurons.

#### Eg5 Inhibitions Suppress VR1 Expression in Axon Terminals and Impair Synapse Formation in the Superficial Laminae of the Spinal Cord

**Fig. 5:**
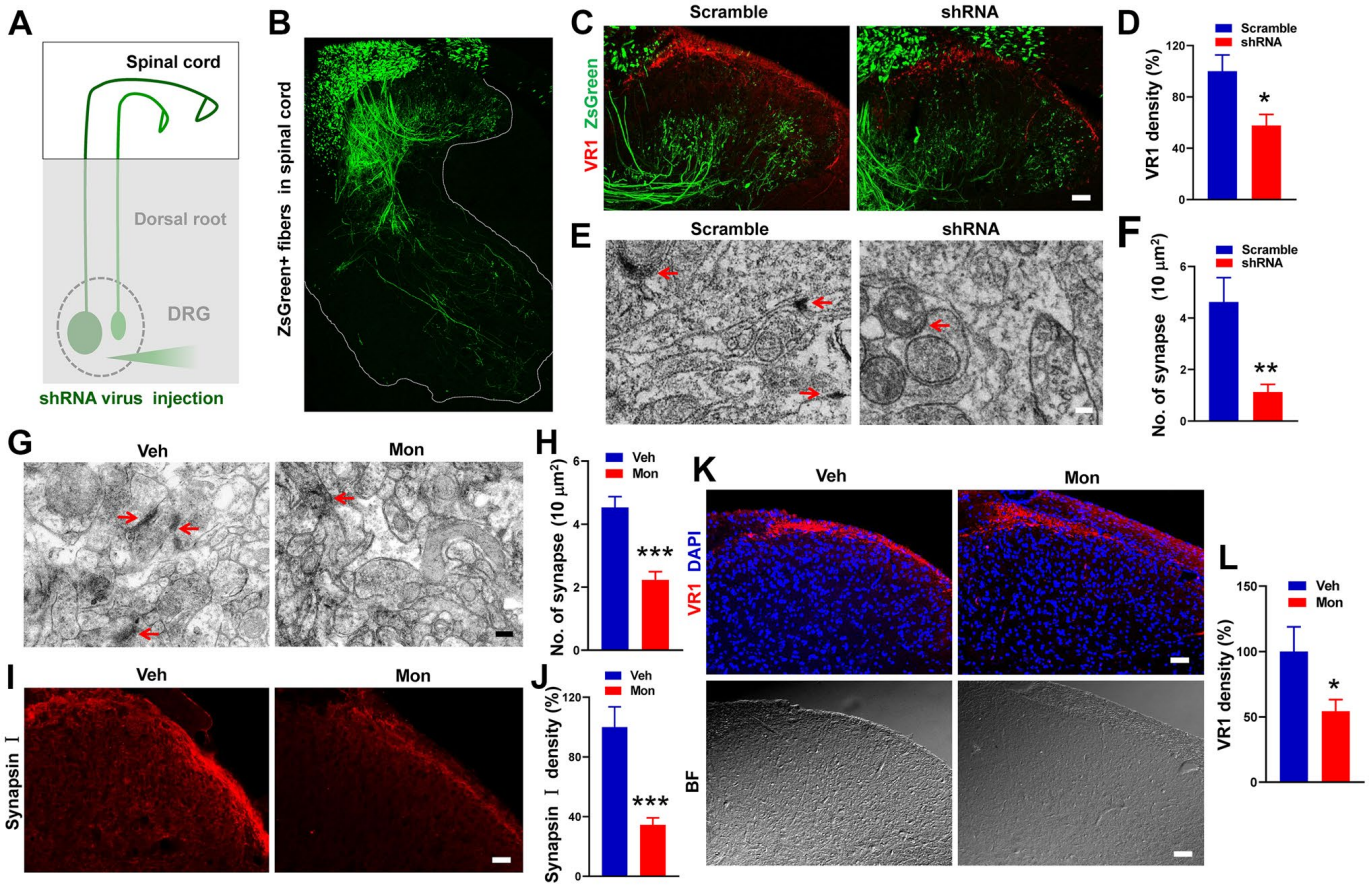
Eg5 inhibition and down-regulation reduce VR1 expression and impair synapse formation in the spinal cord dorsal horn. **A** Schematic of ZsGreen-labelled DRG nerve terminals in the spinal cord after shRNA virus intraganglionic injection. **B** An example of an immunofluorescence image of ZsGreen-positive nerve terminals projecting to the spinal cord. Note that ZsGreen + DRG nerve fibres project to the ipsilateral spinal cord. **C** Examples of immunofluorescence images of VR1 and ZsGreen in the spinal cord dorsal horn with scramble and shRNA. Note that VR1 + terminals are distributed in superficial lamina I/II. **D** Statistical analysis of VR1 density in the spinal cord with scramble and shRNA (*n* = 3 mice and 8–10 sections for each group). **E** Representative electron microscopy images of synapses in spinal cord superficial lamina I/II with scramble and shRNA treatments. Arrows indicate synapses. **F** Statistical analysis of synapse number in 10 μm^2^ in the superficial lamina I/II with scramble and shRNA treatments (*n* = 3 mice and 8 sections for each group). **G** Representative electron microscopy images of synapses in superficial lamina I/II with vehicle and monastrol i.t. treatment. Arrows indicate synapses. **H** Statistical analysis of synapse number in 10 μm.^2^ in the superficial lamina I/II with vehicle and monastrol (*n* = 3 mice and 15 sections for each group). **I** Examples of immunofluorescence images of synapsin I in the spinal cord with vehicle and monastrol i.t. treatment. **J** Statistical analysis of synapsin I density in spinal cord with vehicle and monastrol (*n* = 3 mice and 8–9 sections for each group). **K** Examples of immunofluorescence images of VR1 in the spinal cord dorsal horn with vehicle and monastrol. BF, bright field. **L** Statistical analysis of VR1 density in the spinal cord with vehicle and monastrol i.t. treatment (*n* = 3 mice and 10–11 sections per group). Veh, vehicle control; Mon, monastrol. Scale bars, 50 μm in (**C**), (**I**) and (**K**) and 200 nm in (**E**) and (**J**). Unpaired *t* test in (**D**), (**F**), (**H**), (**J**) and (**L**). *P < 0.05, **P < 0.01, ***P < 0.001. Error bars show the mean ± SEM

We further examined the effects of Eg5 inhibition in the spinal cord. After Eg5 shRNA virus intraganglionic injection (Fig. 5A), we found that ZsGreen + nerve fibres were intensively distributed in the ipsilateral dorsal and ventral spinal cord (Fig. 5B). Eg5 shRNA increased the number of input fibre bundles vertically projected from the ZsGreen + nerve fibres in the dorsal funiculus (12 ± 1.1 versus 22 ± 1.0, *P* < 0.001, Fig. S3A, B) and dorsal horn laminae I/II (9 ± 0.6 versus 21 ± 1.2, *P* < 0.001, Fig. S3A, C). Additionally, Eg5 shRNA promoted axonal branching in cultured DRG neurons (Fig. S4D), supporting the roles of Eg5 in axon growth and development [27]. Immunohistochemistry results showed that Eg5 shRNA intraganglionic injection caused VR1 reduction in the superficial laminae of the spinal cord dorsal horn (100 ± 12.7% versus 57.8 ± 8.5%, *P* < 0.05, Fig. 5C, D).

Electron microscopy results showed a wide distribution of synaptic profiles in superficial lamina I/II but not in deep lamina (Fig. S4). We found that Eg5 shRNA virus suppressed the number of synapses (per 10 μm^2^) in spinal cord laminae I/II (5 ± 1.1 versus 1 ± 0.3, *P* < 0.001, Fig. 5E, F). The effects of Eg5 inhibition on synapses were also detected by monastrol i.t. treatment (5 ± 0.3 versus 2 ± 0.3, *P* < 0.001, Fig. 5G, H). Immunostaining of spinal cord sections showed that synapsin I density was reduced by monastrol i.t. treatment (100.0 ± 1.5% versus 34.5 ± 0.6%, *P* < 0.001, Fig. 5I, J). Monastrol also reduced VR1 expression in the spinal cord (100 ± 2.5% versus 54.3 ± 1.1%, *P* < 0.05, Fig. 5K, L). Moreover, CFA inflammation had no effects on the number of synapses or synapsin I expression in laminae I/II (Fig. S5).

**Fig. 6:**
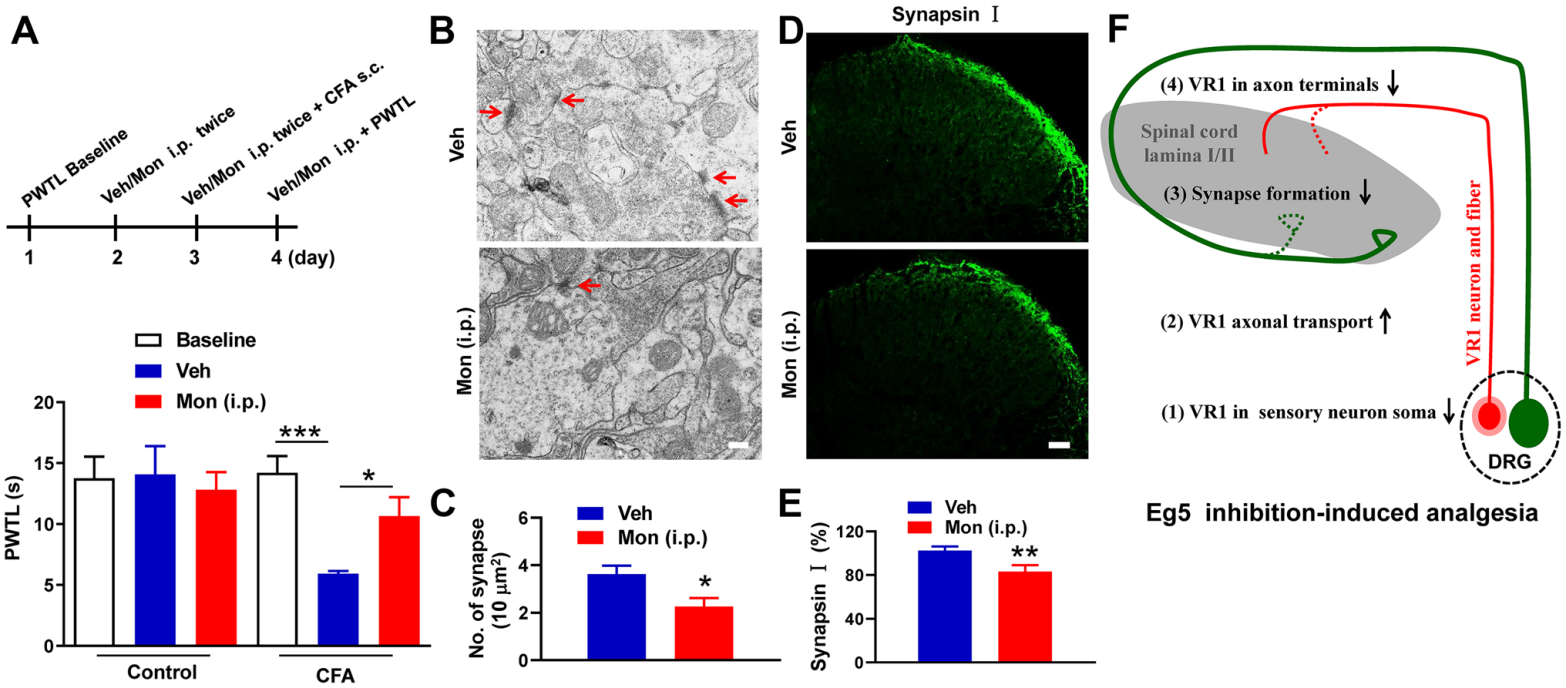
Suppressive effects of monastrol i.p. treatment on pain behaviour and synapse formation in spinal cord. **A** Schematic of monastrol i.p. treatment and the effects on CFA-induced inflammatory pain behaviour (*n* = 8–9 mice for each group). **B** Representative electron microscopy images of synapses in superficial lamina I/II with vehicle and monastrol. Arrows indicate synapses. **C** Statistical analysis of synapse number in 10 μm.^2^ in the superficial lamina I/II with vehicle and monastrol (*n* = 3 mice and 15 sections for each group). **D** Representative immunofluorescence images of synapsin I in the spinal cord with vehicle and monastrol. **E** Statistical analysis of synapsin I density in spinal cord with vehicle and monastrol (*n* = 3 mice and 13–14 sections for each group). **F** Diagram of Eg5 inhibition-induced analgesia on inflammatory pain via multiple mechanisms from peripheral to central levels. Upwards arrow, increase; downwards arrow, decrease. Veh, vehicle control; Mon, monastrol. Scale bars, 200 nm in (**B**) and 50 μm in (**D**). One-way ANOVA with Tukey’s post hoc test in (**A**) and the unpaired *t* test in (**C**) and (**E**). *P < 0.05, **P < 0.01, ***P < 0.001. Error bars show the mean ± SEM

To detect the systemic effects of Eg5 inhibition, we intraperitoneally (i.p.) administered monastrol (1 mM, 10 μl) twice daily for three consecutive days. We showed that monastrol reversed CFA-induced heat hyperalgesia (5.9 ± 0.02 s versus 10.7 ± 0.2 s, *P* < 0.05, Fig. 6A) and suppressed the number of synapses in lumbar spinal cord laminae I/II (4 ± 0.1 versus 2 ± 0.1, *P* < 0.05, Fig. 6B, C). Synapsin I density was also reduced by monastrol i.p. treatment (102.4 ± 0.6% versus 83.2 ± 0.8%, *P* < 0.01, Fig. 6D, E). These results revealed that Eg5 inhibition induced the loss of synapsin, a specific marker maintaining the organization and abundance of vesicles at presynaptic terminals [28], and impaired synapse formation in the spinal cord.

Collectively, our data showed that the motor protein Eg5 regulated VR1 expression in DRG neuronal soma, nerve fibres and central terminals as well as synapse formation in superficial lamina I/II of the dorsal horn (Fig. 6F). Eg5 inhibition generated analgesia at multiple sites in the sensory nervous system, indicating that Eg5 might be a novel molecular target for pathological pain treatment.

#### Eg5 Is Necessary for PI3K/Akt Signalling-Mediated VR1 Membrane Trafficking

**Fig. 7:**
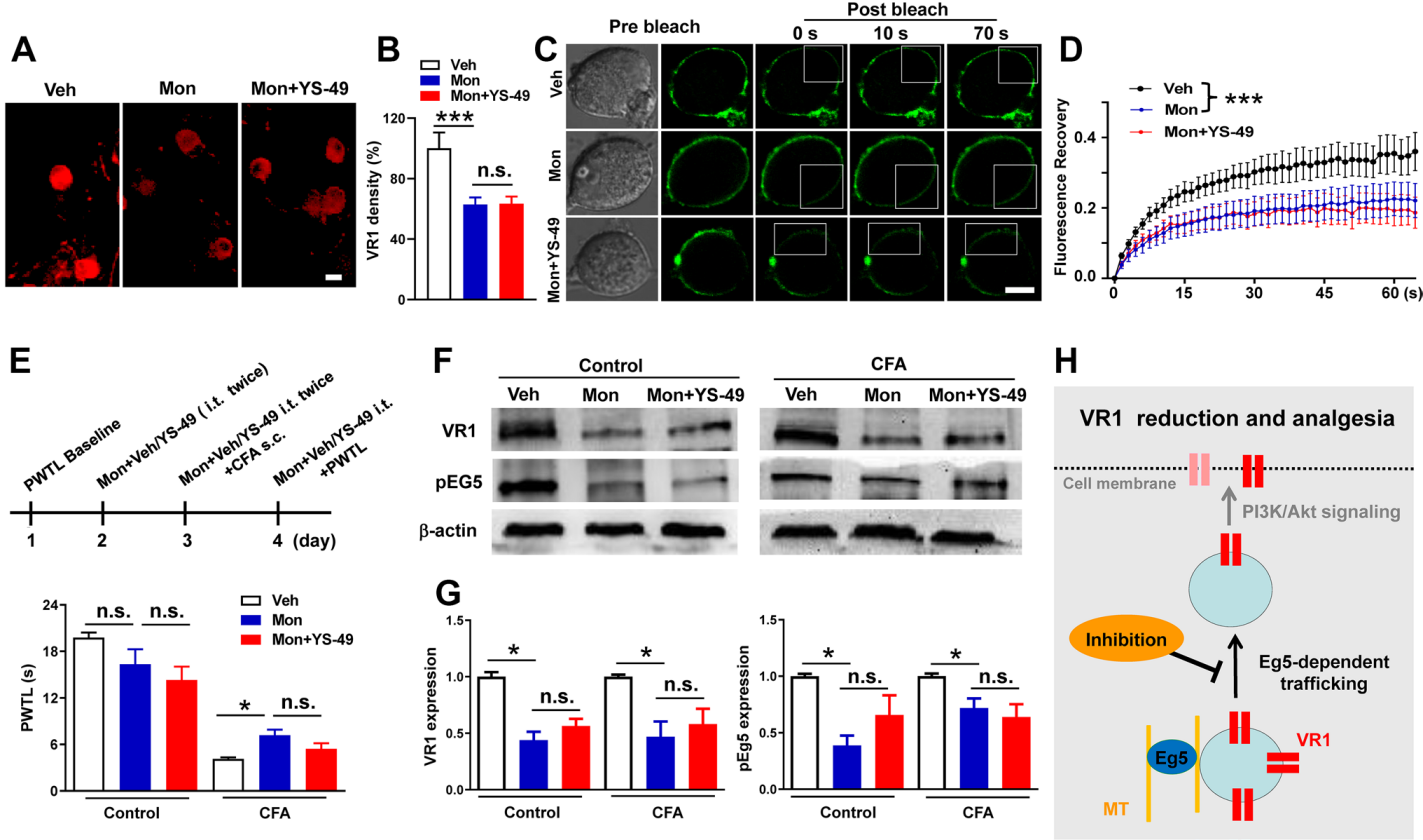
Eg5 is necessary for PI3K/Akt signalling-mediated VR1 membrane trafficking. **A** Examples of immunofluorescence images of VR1 in the culture of DRG neurons with vehicle, monastrol and the PI3K/AKT signalling activator YS-49. **B** Statistical analysis of the VR1 density with vehicle, monastrol and YS-49 (*n* = 3 mice and 63–73 neurons for each group). **C** Representative images of fluorescence recovery of FM1-43 bleaching in the culture of DRG neurons with vehicle, monastrol and YS-49. **D** Statistical analysis of the fluorescence recovery of FM1-43 bleaching with vehicle, monastrol and YS-49 (*n* = 10 neurons for each group). **E** Schematic of monastrol and YS-49 applications and the effects on CFA-induced inflammatory pain behaviour (*n* = 6–11 mice for each group). **F** Representative Western blot of VR1 and pEg5 in the plasma membrane of DRG neurons with vehicle, monastrol and YS-49. **G** Analysis of VR1 and pEg5 proteins in the plasma membrane of DRG neurons treated with vehicle, monastrol or YS-49 (*n* = 4 replicates for each group). **H** Schematic of the Eg5-dependent VR1 membrane trafficking mechanism. Veh, vehicle control; Mon, monastrol; MT, microtubule. Scale bars, 10 μm. One-way ANOVA with Tukey’s post hoc test in (**B**), (**E**) and (**G**). Two-way ANOVA in (**D**). n.s., nonsignificant, *P < 0.05, ***P < 0.001. Error bars show the mean ± SEM

The roles of Eg5 in regulating VR1 expression in neuronal soma, axons and central terminals prompted us to hypothesize that Eg5 might be involved in the process of neuronal membrane trafficking. Phosphoinositide 3-kinase (PI3K)/Akt signalling activation contributes to VR1 cell membrane trafficking and neuron sensitization [29–32]. In the cultured DRG neurons, VR1 density was decreased by monastrol (100 μM), and the reduction was not reversed by the PI3K/Akt signalling activator YS-49 [33] (100 ± 10.4%, 62.8 ± 4.7%, and 63.5 ± 4.7%, *P* < 0.001, Fig. 7A, B). Photobleaching experiments in the plasma membrane of DRG neurons showed that monastrol (100 μM) significantly inhibited FM1-43 (5 μM) fluorescence recovery and that the response was not changed by YS-49 (Fig. 7C, D, Movies S3–S5). These data indicated that Eg5 contributed to PI3K/Akt signalling-mediated VR1 membrane trafficking.

Furthermore, we sought to functionally test whether EG5 and PI3K inhibitions can change the calcium response induced by the VR1 agonist capsaicin. DRG neurons were loaded with the ratiometric indicator fura2-AM (2 μM) for live cell imaging of intracellular calcium levels. Capsaicin (5 μM) was applied followed by 50 mM potassium chloride (KCl) as a positive control response in DRG neurons with different dilutions of vehicle, monastrol (100 μM) and the PI3K inhibitor LY294002 (10 μM) (Fig. S6A). We found that the capsaicin-induced calcium response (44.0 ± 6.66) was significantly reduced by monastrol and LY294002 (14.6 ± 2.04 and 28.2 ± 3.52, *P* < 0.001 and *P* < 0.05, Fig. S6B, C). Additionally, LY294002 decreased VR1 expression in DRG neurons (100 ± 7.2% and 48.7 ± 5.76%, *P* < 0.001, Fig. S6D, E). These results suggested that Eg5 and PI3K inhibitions suppressed VR1 membrane expression and function.

To confirm the roles of Eg5 in VR1 membrane expression, we applied YS-49 i.t. administration (10 μM/10 μl, twice daily for three consecutive days) with or without monastrol (Fig. 7E). Behavioural experiments showed that CFA-induced thermal pain was reduced by monastrol (4.1 ± 0.2 s versus 7.2 ± 0.7 s, *P* < 0.05), and YS-49 failed to reverse this change (Fig. 7E). Western blot results showed that VR1 membrane expression was significantly reduced by monastrol (1.0 ± 0.01 versus 0.5 ± 0.01, *P* < 0.05), and the response was not changed by YS-49. Similarly, YS-49 had no effects on monastrol-induced pEg5 reduction (1.0 ± 0.01 versus 0.7 ± 0.01, *P* < 0.05, Fig. 7F, G). Collectively, our data indicated that Eg5 was necessary for PI3K/Akt signalling-mediated VR1 membrane trafficking (Fig. 7H).

### Discussion

In the present study, we first reported a previously unknown noncanonical function of the motor protein Eg5 in pathological pain. In vivo and in vitro data showed that Eg5 inhibition abrogated PI3K/Akt signalling-mediated VR1 plasma membrane trafficking and reversed pathological pain. Targeting Eg5-dependent membrane trafficking of pain regulators will be a novel strategy for pathological pain treatment.

The VR1 ion channel is essential for the thermal sensation of neurons, and its plasma membrane expression largely dominates the whole-cell response in pain [25, 26]. The process of VR1 membrane trafficking can be modulated by cyclin-dependent kinase 5 (Cdk5), synaptotagmin and synaptic vesicle membrane protein VAMP1 [17, 34–36]. Phosphoinositide 3-kinase (PI3K) interacts directly with the N-terminal region of VR1 and facilitates its trafficking to the plasma membrane [29, 32]. Activation of the PI3K/Akt signalling pathway contributes to pathological pain [30, 31]. In the present study, we further showed that Eg5 was upstream of PI3K/Akt signalling-mediated VR1 plasma trafficking. Pharmacological inhibition and shRNA knockdown impaired VR1 trafficking and reversed inflammatory pain behaviour.

The contribution of VR1 axonal transport in nerve fibres to pain was reported two decades ago [37, 38]. However, the molecules mediating the process remain unclear. Previous data showed that Eg5 inhibition enhanced the transport frequency of short microtubules but not rhodamine-dextran-labelled vesicles and FM-labelled mitochondria [12]. Here, we found that Eg5 inhibition could speed up the anterograde transport of ZsGreen fluorescence protein in axons. Furthermore, by using the VR1-mCherry virus, we revealed that Eg5 inhibition accelerated the average velocity of VR1 anterograde transport and abrogated VR1 expression in the axons of DRG neurons. Our results indicate that Eg5 is the key molecule modulating VR1 axonal transport in sensory nerve fibres.

In the present study, we found that local and systemic applications of monastrol and shRNA virus suppressed synapse formation in the superficial laminae of the spinal cord. Eg5 inhibition-induced synapse impairment was unexpected. One interesting finding was that monastrol caused dendritic spine loss in cultured mouse hippocampal neurons [39]. Here, we showed that the loss of synapsin, the specific protein maintaining synapse organization [28], was responsible for synapse impairment in the spinal cord. FM1-43 photobleaching tests showed that Eg5 inhibition abrogated fluorescence recovery, suggesting the roles of Eg5 in maintaining membrane fluidity. Membrane fluidity contributes to the formation and function of synapses [40]. Therefore, membrane fluidity perturbation induced by Eg5 inhibition contributed to synapse impairment. Additionally, synapse maturation requires a high amount of energy [41] and is involved in a series of steps from neurite formation, outgrowth and branching in the early stage to synapse formation, maturation and plasticity in the late stage [42]. Our results suggested that, under Eg5 inhibition, the increased number of input fibre bundles and axonal branching might not efficiently support synapse formation. Our study provide some reasonable explanations for the roles of Eg5 in synapse formation in the spinal cord.

The origins of synapse connections in the spinal cord are tremendously complex, containing nerve fibre terminals from primary DRG neurons, local excitatory and inhibitory interneurons and descending GABAergic or serotonergic neurons from the brain rostroventral medulla [43]. We found that Eg5 shRNA virus intraganglionic injection decreased the number of synapses in superficial laminae, indicating that the decreased synapses were arising from primary DRG neurons.

Spinal cord superficial laminae received afferent projections of thinly myelinated Aδ, non-myelinated C-fibres and even heavily myelinated Aβ fibres coming from DRG neurons [44–49]. By the ZsGreen + virus transfection approach, we found that large-sized DRG neurons projected axonal fibres crossing the deep laminae and finally terminated in superficial laminae. Therefore, the synapse originating from Aβ, Aδ or C fibre that is the target of Eg5 still needs to be clarified. Additionally, Eg5 is widely expressed in small-, medium- and large-sized DRG neurons, transmitting diverse somatosensory information coming from proprioceptors, mechanoreceptors, thermoceptors and chemoreceptors [46–48]. It will be of interest to determine the potential functions of motor protein Eg5 in other types of somatic sensory, such as itch and touch.

### Conclusions

In the present study, our data show that Eg5 regulates VR1 expression in DRG neuronal soma, nerve fibres and central terminals as well as synapse formation in the superficial lamina of the spinal cord. We further identify a novel molecular mechanism by which Eg5 contributes to pathological pain and VR1 membrane trafficking through the PI3K/Akt signalling pathway. These findings lay the groundwork for targeting Eg5 in mature postmitotic neurons in pathological pain modulation. Eg5 inhibition might be an efficient strategy to alleviate intractable pain.

## Supplementary Information

Below is the link to the electronic supplementary material.

Supplementary file1 (PDF 57 KB)

Supplementary file2 (PDF 509 KB)

Supplementary file3 (PDF 517 KB)

Supplementary file4 (PDF 526 KB)

Supplementary file5 (PDF 535 KB)

Supplementary file6 (PDF 543 KB)

Supplementary file7 (PDF 552 KB)

Supplementary file8 (MOV 1434 KB)

Supplementary file9 (MOV 1241 KB)

Supplementary file10 (MOV 642 KB)

Supplementary file11 (MOV 503 KB)

Supplementary file12 (MOV 493 KB)

Supplementary file13 (TIF 7271 KB)

Supplementary file14 (TIF 6925 KB)

Supplementary file15 (TIF 6792 KB)

Supplementary file16 (TIF 11198 KB)

Supplementary file17 (TIF 9854 KB)

Supplementary file18 (TIF 5004 KB)
